# Pseudomyxoma Peritonei Secondary to a Primary Appendix Tumor: A Belly Full of Jelly

**DOI:** 10.7759/cureus.5221

**Published:** 2019-07-24

**Authors:** Adebola O Sorungbe, Emily Whiles, Elisabeth Drye, Samson O Oyibo

**Affiliations:** 1 Diabetes and Endocrinology, Peterborough City Hospital, Peterborough, GBR; 2 General Surgery, Peterborough City Hospital, Peterborough, GBR

**Keywords:** pseudomyxoma peritonei, ascites, tumor markers, cytoreductive surgery

## Abstract

Pseudomyxoma peritonei (PMP) is a rare and life-threatening cancer of the abdominal peritoneum. Symptoms can be non-specific and ignorable for several years such that most cases are diagnosed during explorative surgery. The cornerstones of diagnosis and effective management are heightened awareness and clinical suspicion, timely recognition, and early referral to a specialist center for work-up and cytoreductive surgery. We report an interesting case of a patient with PMP who had successful cytoreductive surgery and heated intraperitoneal chemotherapy.

## Introduction

Peudomyxoma peritonei (PMP) is a poorly understood and rare tumor of the abdominal peritoneum. The diseased peritoneum produces an excessive amount of mucinous fluid which gradually fills the peritoneal cavity to the point of compressing vital organs, such as the pancreas, spleen, stomach, kidneys, liver, and the colon [1]. The estimated incidence of PMP gathered from clinical caseload experience at national specialist centers is 1-2 per million per year [1]. We report an interesting case of a patient with PMP who had successful cytoreductive surgery and heated intraperitoneal chemotherapy (HIPEC).

## Case presentation

A 69-year-old man attended the emergency department having had severe acute onset left-sided abdominal pain. The pain had resolved before attendance. Further history taking revealed that he had noticed increasing abdominal girth over the previous 6 months. He had no past medical history of note and was not on any regular medication. He was a non-smoker.

On physical examination, the patient appeared generally well. He was hemodynamically stable and apyrexial. Abdominal examination revealed a soft non-tender abdomen.

Blood results demonstrated mild normocytic anemia but normal liver and kidney function. The C-reactive protein was slightly raised (Table 1).

**Table 1:** Patient’s blood results with laboratory reference values

Blood parameters	Normal values	Patient’s results
Hemoglobin (g/dl)	130-180	121
Mean cell volume (fl)	80-100	81.1
White cell (10^9^/L)	4-11	10.3
Platelets (10^9^/L)	150-400	418
Sodium (mmol/L)	133-146	135
Potassium (mmol/l)	3.5-5.3	5.2
Creatinine (µmol/L)	59-104	61
Albumin (g/L)	35-50	39
Bilirubin (µmol/L)	<21	8
Alkaline phosphatase (U/L)	30-130	96
Glucose (mmol/l)	<7	6.2
C-reactive protein (mg/L)	<10	93
Lactate (mmol/L)	0.6-2.5	0.9
Amylase (U/L)	0-100	45

**Figure 1:**
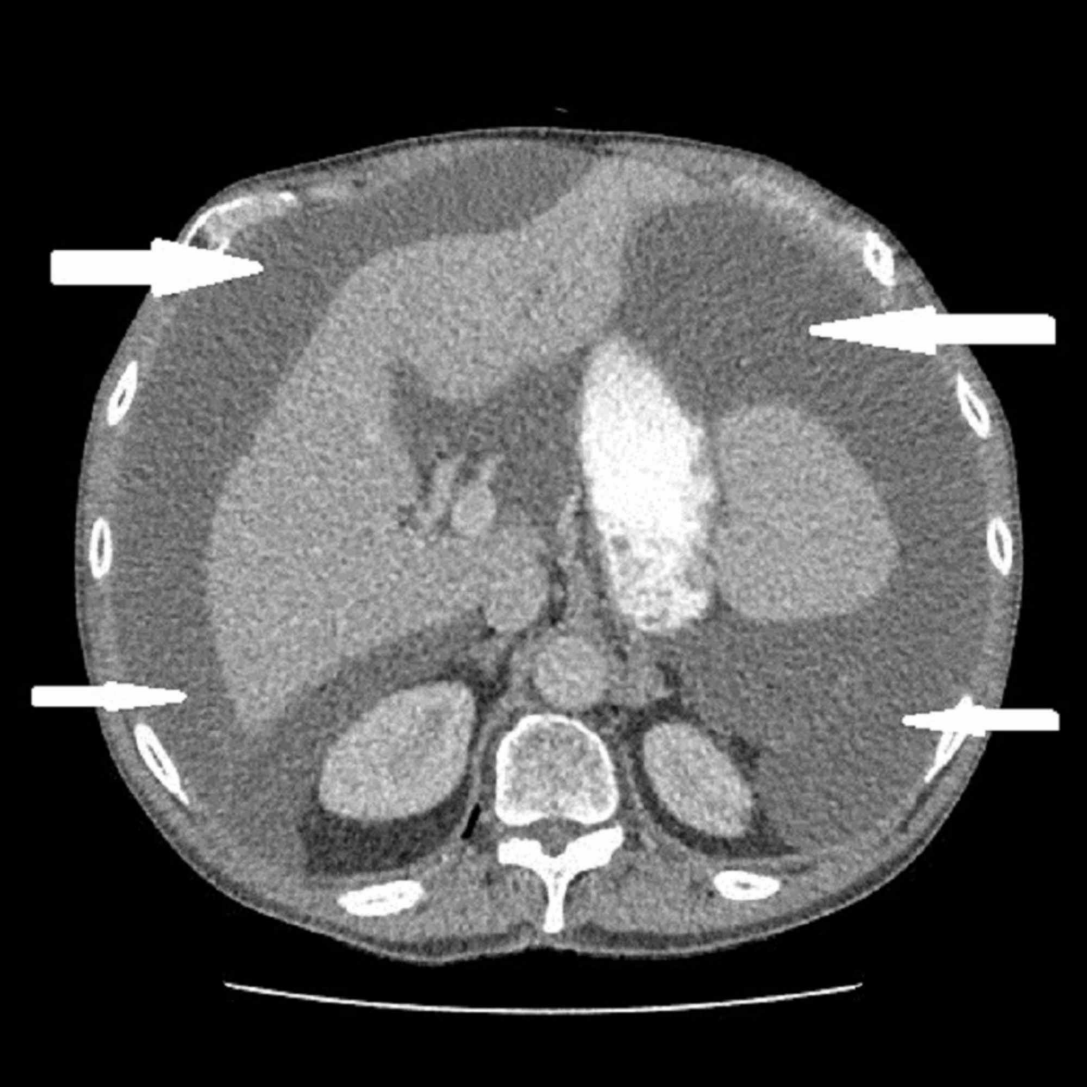
Computerised tomography scan of abdomen showing large ascites (arrows)

A chest and abdominal x-ray did not reveal any abnormal findings. A computerised tomography scan revealed a large amount of free fluid within the peritoneal cavity with high attenuation (20-27 Hounsfield Units) (Figure 1).

**Figure 2:**
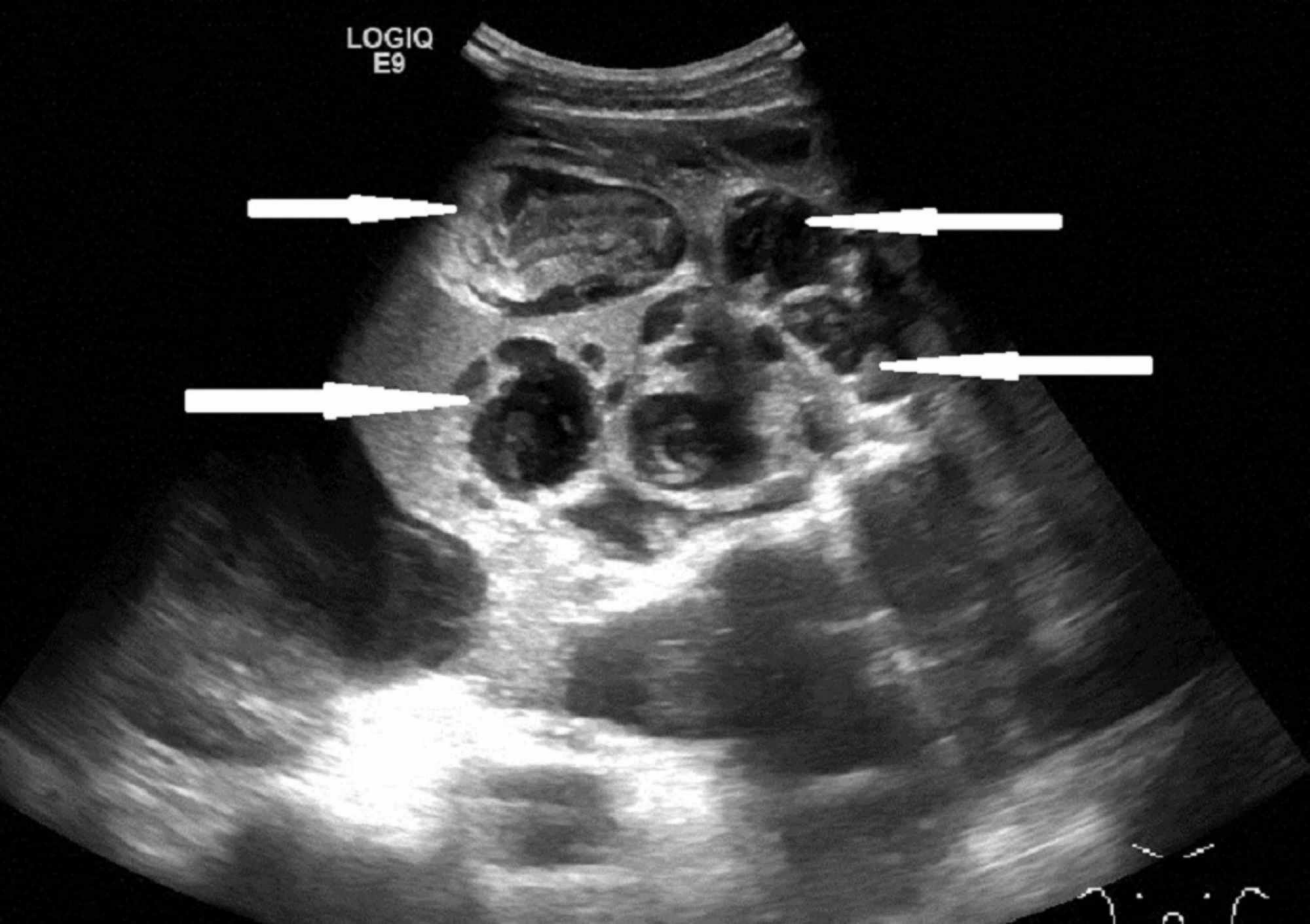
Ultrasound of abdomen showing multiloculated ascites with extensive internal debris (arrows)

A follow-up ultrasound scan of his abdomen revealed extensive multiloculated ascites which contained extensive internal debris. The appearances were suggestive of pseudomyoxma peritonei (Figure 2).

Tumor markers results indicated raised carcinoembryonic antigen (CEA) and cancer antigen 19.9 (CA19.9) levels (Table 2).

**Table 2:** Results of tumor markers with laboratory reference values

Tumors markers	Normal values	Patient’s results
Carcinoembryonic antigen CEA (µg/L)	<4	24
Cancer antigen CA19.9 (U/ml)	<33	42

Laparotomy revealed large volumes of multiple cystic collections of mucinous material from a perforated primary appendix tumor. The liver, spleen, and part of the large bowel were affected (scalloping) and the small bowel serosal surfaces were lightly coated with mucin. The patient underwent complete cytoreductive surgery, appendectomy, splenectomy, and HIPEC (peritoneal cavity perfusion of Mitomycin-C at 42 degrees centigrade for one hour). A histological diagnosis of low-grade appendiceal mucinous neoplasm with low-grade mucinous carcinoma peritonei was made. The patient is being followed up with yearly scans.

## Discussion

Pseudomyxoma peritonei was first described in 1842 by Karl Rokitansky and later described by Werth in association with a mucinous carcinoma of the ovary in 1884 and subsequently described by Frankel in association with an appendiceal cystic tumor in 1901 [1].

Patients with PMP are usually asymptomatic for many years before diagnosis but the commonest symptoms are abdominal distension or increase in abdominal girth with non-specific abdominal pain during the advanced stages of the disease. Other symptoms and signs depend on organ involvement or compression.

Computed tomography (CT) scan is the best imaging technique for diagnosis and staging. The pathognomonic feature is the appearance of areas of low attenuation with islands of high attenuation due to solid elements within mucinous material. Scalloping of visceral surfaces especially liver and spleen distinguish this from fluid ascites [1]. The tumor markers, e.g., CEA, cancer antigen 19.9 (CA19.9), and cancer antigen 125 (CA125) can be used for follow-up and also as prognostic markers. Paracentesis or laparoscopy and biopsy are required for histological confirmation of the presence of mucinous neoplastic cells/epithelium, mucinous ascites, and diffuse mucinous implants [1]. The histological classification of PMP is based on the understanding of the histogenesis, molecular genetic findings and clinical behavior [2-3].

Optimal treatment involves a combination of complete tumor excision by complex surgical peritonectomy (cytoreductive surgery) and HIPEC using cytotoxic medication to target residual microscopic disease [1].

Acute abdominal pain has been a noted symptom in a fifth of cases of PMP [4]. The patient in this case was asymptomatic for several years until he presented with acute abdominal pain which had resolved before attending the emergency department. It was only in hindsight that he noticed increased abdominal girth. The CT scan was arranged because he had accompanying mildly raised C-reactive protein levels, and the ultrasound was arranged because of the presence of mild splenomegaly. This case emphasizes the ease with which a diagnosis of PMP can be missed.

## Conclusions

In conclusion, we have described a patient who developed pseudomyxoma peritonei in the setting of a perforated appendiceal tumor. The presentation was acute with the only symptom being abdominal pain. As with other cases, the diagnosis was made histologically after surgical treatment. The cornerstones of diagnosis and effective management are heightened awareness and clinical suspicion, timely recognition, and early referral to a specialist center for work-up and cytoreductive surgery. We hope that this case will not only add to the existing literature but also contribute to the characterization and management of this rare disease.
